# Infant Feeding

**Published:** 1905-12-23

**Authors:** 


### INFANT FEEDING.

The October issue of the *Practitioner* is devoted to the disorders and management of infancy. The best methods of artificially rearing infants who for one reason or another are denied the nourishment natural to their time of life is well dealt with by Dr. Cautley. So much has been written upon this subject that the essentials of success are not infrequently obscured by a maze of subtleties. There is no need for precise and pedantic imitation of Nature in the provision of an artificial substitute for breast milk, as some would have us believe, but the fundamental differences in the composition of human and cow's milk require to be intelligently borne in mind.

The index of successful hand-feeding is the increase in body-weight, associated, of course, with evidence of physical ease and contentment. This gain is variable in a state of health. Dr. Cautley estimates it at five to seven ounces a week during the first three months, four to six ounces a week during the second three months, and three to five ounces a week during the third three months.

Fresh milk is essential, for the older it is the more likely is it to be contaminated. The practice of adding preservatives to milk is highly to be condemned, for they impart a sense of false security. Milk so treated may not be sour, for the multiplication of the lactic acid bacillus is inhibited easily; yet it may contain organisms much more potent for evil, which are unaffected by the small proportions of such antiseptics as are in common use. The souring of milk is therefore a valuable indication of unsuitability as regards infant feeding at all events, and any process which tends to obscure this indication is a danger to infant life.

It is recommended that the milk should be obtained twice daily from a big dairy company of good repute, and placed in a tall, narrow vessel in a cool place for two or three hours. The upper half is then to be syphoned off and kept for use; the bottom milk is likely to contain much gross contamination, and should be applied to some other purpose than infant feeding.

Dr. Cautley suggests that for the sake of ease in calculation the following percentages of the various constituents of human milk and cow's milk respectively may be accepted as sufficiently accurate:—

Human milk ... ... Proteid 2, Fat 4, Sugar 6.6.
Cow's milk ... ... Proteid 4, Fat 4, Sugar 4.

A further difference of importance lies in the fact that in human milk the caseinogen bears to the lactalbumin a proportion of about one to two, while in cow's milk the proportion is about four to one. A consideration of these variations makes it clear that no simple dilution of cow's milk can provide a fluid containing the various elements of human milk in anything like their proper proportions, for a dilution which will reduce the proteid ingredients to a sufficient extent will leave the resulting fluid far too poor in fats and sugar.

In securing an appropriate diet for an infant, it is wise to begin with a weak mixture. Dr. Cautley recommends the following procedure: The top milk obtained, as indicated above, will contain the cream of the whole, and may therefore be considered to contain twice the ordinary fat-value of cow's milk, or approximately 8 per cent., and the usual values of proteid and sugar. Such milk diluted with an equal bulk of water will contain approximately correct values of proteid and fat, but will require the addition of some 4 per cent. of sugar. It will then conform with reasonable accuracy with the composition of human milk. For an infant under the age of three months the initial dietary ought to be rather weaker in proteid constituents than this, and Dr. Cautley suggests the following as a good formula for the inception of hand feeding at this age: Top milk, 5 ounces; lime-water, 1 ounce; sugar, 6½ drachms; and water, 14 ounces. The mixture will then contain proteid, 1 per cent.; fat, 2 per cent.; sugar, 5 per cent.

As regards the boiling of milk, the author considers that heat impairs its value as a food, and that therefore boiling is contra-indicated if the quality of the milk is guaranteed and if proper intelligence in its treatment can be secured. Otherwise it is well to boil it.

The reaction of human milk is to be regarded as alkaline, although it gives a faintly acid reaction with phenolphthalein; cow's milk, on the other hand, is strongly acid by the time it reaches the baby. The addition of an alkali makes the milk more analogous to human milk, and, by neutralising the acid, renders it much less firmly coagulable by rennet. Lactic acid leads to the formation of tough curds in the stomach, and is a frequent source of gastric dyspepsia. This firm curdling is prevented by alkalies, but they must not be given in quantities large enough to inhibit the action of the rennet.

Infants should be fed at regular intervals, and must, if necessary, be roused from sleep for this purpose during the early months of life. Ten feeds at intervals of two hours during the first month, eight feeds at intervals of two and a half hours during the second month, and seven feeds at intervals of three hours thereafter until the conclusion of bottle feeding. It may be possible to enlarge the interval and the quantities after the sixth month if the child is doing well. As regards quantity no definite rule can be laid down, but the author suggests the following table for the first month of life. The quantities are given in drachms.

| *Week of Life.* | *One* | *Two* | *Three* | *Four* |
|---|---|---|---|---|
| Milk ... ... ... | 2 | 3 | 4 | 5 |
| Cream ... ... | 1 | 1 | 1 | 1 |
| Water ... ... | 5 | 6 | 7 | 8 |
| Lime water ... ... | 1 | 1 | 1 | 1 |
| Sugar ... ... | ½ | ½ | ½ | ½ |

As regards the variety of sugar to be used, Dr. Cautley considers that undoubtedly milk-sugar is the best, but that there is no evidence that cane-sugar is harmful if used in moderation.

